# Influence of thickness and base material in class II restorations with nanofilled composites: finite element study

**DOI:** 10.3389/fdmed.2026.1717959

**Published:** 2026-02-13

**Authors:** Fredy Hugo Cruzado-Oliva, Alexander Vega-Anticona, David Arturo Ortiz-Diaz, Heber Isac Arbildo-Vega, Franz Tito Coronel-Zubiate

**Affiliations:** 1 Escuela de Estomatología, Facultad de Estomatología, Universidad Nacional de Trujillo, Trujillo, Perú; 2 Faculty of Engineering, School of Materials Engineering, Universidad Nacional de Trujillo, Trujillo, Peru; 3 Faculty of Dentistry, School of Dentistry, Faculty of Human Medicine, School of Human Medicine, Universidad San Martín de Porres, Chiclayo, Peru; 4 Posgraduate School, Universidad Nacional Toribio Rodríguez de Mendoza de Amazonas, Chachapoyas, Perú; 5 Faculty of Health Sciences, Stomatology School, Universidad Nacional Toribio Rodríguez de Mendoza de Amazonas, Chachapoyas, Perú

**Keywords:** base material, base thickness, biomechanics, composite resin restorations, dental restoration, finite element analysis

## Abstract

**Statement of problem:**

The selection of restorative and base materials in the restoration of large cavity preparations remains a debatable issue, and it is unclear how functional stresses affect Class II (MOD) restorations.

**Objective:**

To evaluate the influence of base material type and thickness on stress distribution in Class II MOD restorations using various nanofilled packable composites.

**Material and methods:**

Eight three-dimensional finite element models were developed: six experimental models combining three restorative composites (Filtek Supreme, Grandio and Admira Fusion) with two base materials [flowable resin composite [FR] and resin-modified glass ionomer [RMGI]] at thicknesses of 0.5 mm, 1.0 mm, and 1.5 mm; and two control models, including restorations without a base layer and a healthy, intact tooth. A static vertical load of 600 N was applied along the tooth's long axis, and Von Mises stress distribution was analyzed within the base, restoration, and dental structures.

**Results:**

The sound tooth model exhibited the lowest stress concentration (20.777 MPa). Among the restored groups, models without a base layer showed higher stress values, with Filtek Supreme XTE reaching a maximum of 31.243 MPa. Overall, the incorporation of base materials improved stress distribution. The most favorable outcomes among the restored models were observed with the combination of Grandio and Fuji II GC glass ionomer, which yielded the lowest stress value (20.846 MPa). In contrast, the use of 1.5 mm-thick flowable resin bases tended to increase stress levels (up to 32.031 MPa in the Admira Fusion models) compared with thinner layers or glass ionomer alternatives.

**Conclusion:**

Thicker resin-modified glass ionomer (RMGI) bases provide a more favorable stress distribution than flowable resin composite (FR) bases in Class II restorations.

## 1 Introduction

Dental caries remains one of the most widespread chronic conditions globally (1), significantly impacting the oral health of adults (2). The clinical management of large carious lesions continues to be a challenge, particularly when selecting the most effective treatment that ensures long-term success. Historically, caries management involved non-selective removal of all infected and affected dentin to halt the progression of disease, followed by restoration using a suitable material (3).

Composite resins have become the predominant restorative material in current practice due to their favorable aesthetic properties, conservative nature, ease of manipulation, and enhanced mechanical characteristics. These attributes make them the material of choice for posterior restorations subjected to high occlusal forces, especially those involving the marginal ridge (4).

Despite these advantages, one of the main drawbacks of composite resins is polymerization shrinkage, which can lead to debonding from the tooth structure (5–7). This issue is particularly critical at the gingival floor of Class II cavities, where achieving an optimal marginal seal is more difficult (8). Inadequate sealing at the interface may allow for microleakage, bacterial infiltration, postoperative sensitivity, secondary caries, pulpal irritation, and eventual failure of the restoration (9, 10).

To reduce the stresses associated with polymerization shrinkage, a layered approach—commonly known as the sandwich or double-laminate technique—has been recommended (11). This method incorporates an intermediate layer with distinct elastic deformation properties, followed by the composite restorative material (12–14). The intermediate base serves as a stress-absorbing barrier, mitigating the contraction forces during polymerization, while the overlying composite restores mechanical integrity and esthetics (15–18).

Among the materials used as base liners, resin-modified glass ionomer (RMGI) cements offer chemical adhesion to dentin, micromechanical bonding to composite resin, pulpal protection, potential anticariogenic activity, and reduced polymerization shrinkage and microleakage (15, 19). Alternatively, flowable resin composites (FRs) have gained acceptance due to their low viscosity, which enhances adaptation to cavity walls and reduces microleakage (19, 20). Their low elastic modulus also helps dissipate stress, while their adhesive properties contribute to reinforcement and durability in subsequent restorations (4, 21).

Although randomized clinical trials offer high levels of evidence regarding the clinical performance of Class II restorations, such studies are resource-intensive, complex to conduct, and time-consuming. As a result, *in vitro* testing methods remain valuable for comparing materials under standardized conditions. However, traditional laboratory methods are often destructive and may not detect early crack propagation or subtle stress distributions (22). Finite element analysis (FEA), by contrast, is a well-established computational tool that allows for non-invasive evaluation of internal stresses and strains. In dentistry, FEA has proven particularly useful in analyzing the biomechanical behavior of restored teeth and identifying stress concentration zones at the tooth-restoration interface (23–25).

Selecting an appropriate combination of base and restorative materials is essential to reduce the negative consequences of polymerization shrinkage in Class II restorations. However, there is no consensus regarding the ideal materials or optimal base thickness to be used in such cases. Furthermore, the available evidence on how these variables influence stress distribution within the restored structure remains limited.

Accordingly, the present study aimed to assess the effect of two base materials—resin-modified glass ionomer (RMGI) and flowable resin composite (FR)—at different thicknesses (0.5 mm, 1 mm, and 1.5 mm) on stress distribution in Class II mesio-occluso-distal (MOD) restorations using various nanofilled composite resins. The null hypothesis was that no difference in stress distribution would be found among the various designs under identical loading conditions.

## 2 Materials and methods

### 2.1 Study design

This study employed an experimental, cross-sectional, and comparative *in vitro* design based on finite element analysis (FEA). The independent variables consisted of MOD cavity restorations using different combinations of packable composite resins and base materials applied at various thicknesses (0.5 mm, 1 mm, and 1.5 mm). Two additional control groups were included: one featuring a MOD restoration with a packable composite resin only (no base layer), and another representing an unaltered, healthy tooth model (26).

### 2.2 Construction of solid and finite element models

A recently extracted mandibular first molar was selected for modeling, with the following anatomical dimensions: 8 mm crown height, 11 mm mesiodistal width, 10.5 mm buccolingual width, and 13 mm root length. The extraction was performed for periodontal reasons. To digitize the specimen, all surfaces were scanned using a UP560 multifunctional 3D dental scanner (3DBiotech, China). The resulting STL file was refined in Meshmixer software to smooth the surface geometry and reduce polygon count, enhancing model uniformity. The processed model was then exported in SLDDRW format and imported into SOLIDWORKS for solid modeling (22).

Within SOLIDWORKS, the tooth's anatomical plans were defined: *X*-axis (mesial), *Y*-axis (longitudinal), and Z-axis (buccal). Sectioning cuts were applied along these plans to isolate and generate a solid tooth structure suitable for further modeling.

**Figure 1:**
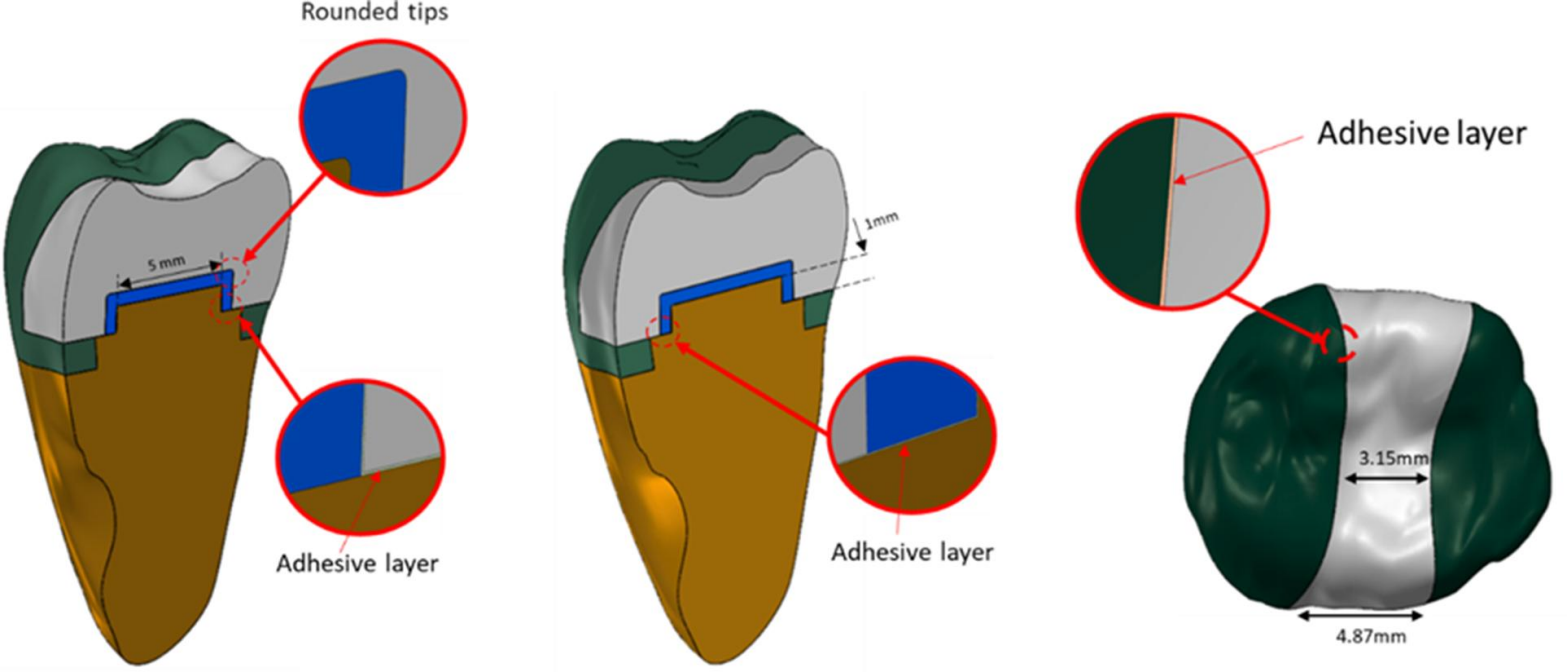
Dimensions of the models.

The MOD cavity, adhesive interface, base materials, and restorative layers were then digitally constructed. Cavity preparation involved an occlusal cut at a depth of 3 mm from the central fissure, with a buccolingual span of 3.15 mm (approximately one-third of the tooth's total buccolingual dimension). The mesial and distal proximal boxes were defined with a width of 4.87 mm. Additionally, a proximal box was designed with 5 mm mesiodistal length, spaced 2.5 mm from both the mesial and distal axial walls, and a 1 mm axial wall thickness. All internal axiopulpal angles were rounded. Across all study models, the cavity walls were constructed at a 95° angle to the axial plane to standardize geometric parameters (26, 27) (Figure 1).

**Figure 2:**
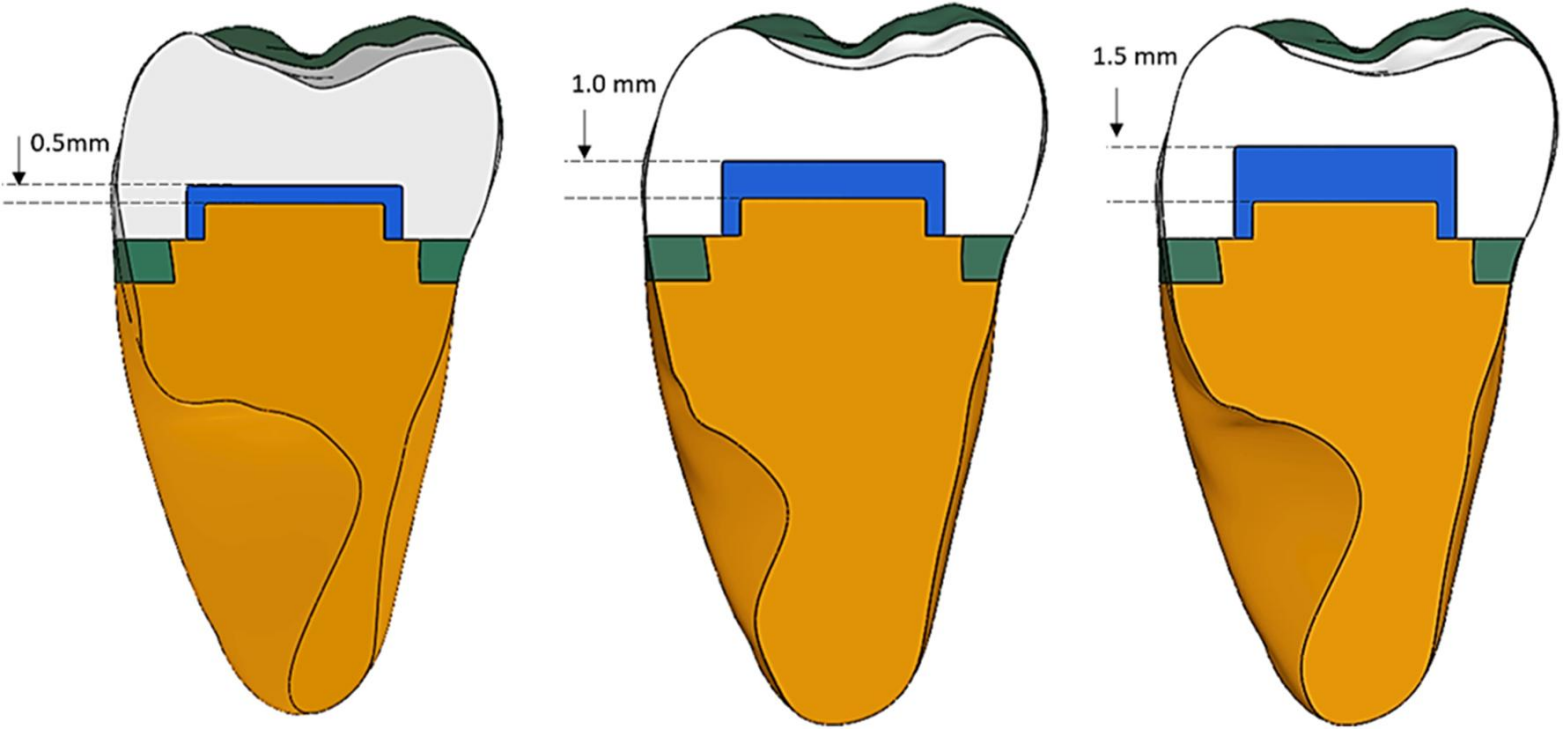
Design of the experimental groups.

The restoration models were developed based on the standardized cavity design. The base materials—either resin-modified glass ionomer (RMGI) or flowable resin composite (FR)—were digitally placed along the pulpal floor at three distinct thicknesses: 0.5 mm, 1.0 mm, and 1.5 mm. Additionally, a uniform 0.5 mm layer of the base material was applied to the axial walls of the pulpal chamber in each experimental model to simulate clinical adaptation (Figure 2).

The finite element mesh was generated using 3D quadratic tetrahedral elements (10-node elements) for all components. A mesh refinement strategy was applied in regions of interest, particularly at the dentin–restoration interface and along the pulpal floor, to capture local stress concentrations (5).

The finalized 3D models were imported into ANSYS simulation software for finite element analysis. The corresponding nodal and element distributions for each model are summarized in Table 1.

**Table 1:** Number of nodes and elements of the different models analyzed using the finite element method.

Analysed models	Base layer thickness (mm)	Number of nodes	Number of elements
Healthy tooth (HT)	None	398633	200799
Restoration with Grandio (G)	None	436688	221359
Restoration with Filtek Supreme (FS)	None	438952	222877
Restoration with admira fusion (AF)	None	439948	223641
Restoration with Grandio + Grandio Flow	0.5	501074	254679
	1.0	502882	255715
	1.5	504890	256948
Restoration with Filtek + Filtek Flow	0.5	501128	254203
	1.0	502304	255842
	1.5	503901	256117
Restoration with Admira + Admira Flow	0.5	501520	254781
	1.0	502376	255109
	1.5	504219	256954
Restoration with Grandio + Fuji II GC glass ionomer	0.5	500050	254226
	1.0	500809	254848
	1.5	501188	255104
Restauration with Filtek + Fuji II GC glass ionomer	0.5	500616	254309
	1.0	501094	254975
	1.5	501944	255148
Restauration with Admira + Fuji II GC glass ionomer	0.5	500079	254112
	1.0	501049	254780
	1.5	501765	255366

### 2.3 Model and group design

The MOD cavity models were digitally segmented into six experimental groups and four control groups. The experimental conditions involved combinations of various nanocomposite restorative materials and base types applied at different thicknesses. The characteristics of the materials used in this study are described in the Table 2.

**Table 2:** Composition and filler characteristics of the restorative and adhesive materials.

Material	Material category	Matrix composition	Filler type	Filler content (wt%/vol%)	Manufacturer
Grandio	Nanohybrid universal resin composite	Bis-GMA, TEGDMA, HEDMA	Silicate glass fillers and nanofillers	∼87 wt%/∼71 vol%	VOCO GmbH, Cuxhaven, Germany
Grandio flow	Nanohybrid flowable resin composite	Bis-GMA, UDMA, HEDMA	Vitroceramic nanoparticles	∼65.6 wt%/∼47 vol%	VOCO GmbH, Cuxhaven, Germany
Filtek supreme XTE	Nanocomposite resin	Bis-GMA, UDMA, TEGDMA, Bis-EMA	Non-agglomerated zirconia nanoparticles (4–11 nm) and aggregated zirconia/silica nanoclusters	∼78.5 wt%/∼63.3 vol%	3M ESPE, St Paul, MN, USA
Filtek supreme flow	Nanocomposite flowable resin	Bis-GMA, TEGDMA, Procrylat	Silica and zirconia nanofillers and zirconia/silica nanoclusters	∼65 wt%/∼55.5 vol%	3M ESPE, St Paul, MN, USA
Admira fusion	Ormocer-based nanohybrid resin composite	Ormocer (organically modified ceramic) matrix	Silicon oxide and vitroceramic fillers	∼84 wt%/∼60 vol%	VOCO GmbH, Cuxhaven, Germany
Admire fusion flow	Ormocer-based nanohybrid flowable resin composite	Ormocer (organically modified ceramic) matrix	Silicon oxide and vitroceramic fillers	∼74 wt%/∼50 vol%	VOCO GmbH, Cuxhaven, Germany
Fuji II GC	Resin-reinforced glass ionomer cement	Polyalkenoic acid, HEMA, water	Fluoroaluminosilicate glass particles	∼70 wt%/∼45–50 vol%	GC Corporation, Tokyo, Japan
Clearfil SE	Two-step self-etch adhesive	10-MDP, Bis-GMA, HEMA, hydrophilic dimethacrylates	Colloidal silica (nanofillers)	∼10 wt%/∼5 vol%	Kuraray Noritake Dental Inc., Tokyo, Japan

Control models included teeth restored without a base layer as well as an anatomically intact, non-restored tooth (26).

### 2.4 Experimental groups

Each experimental group consisted of a tooth restored using a packable nanocomposite resin in combination with a base material [either flowable resin composite [FR] or resin-modified glass ionomer [RMGI]] at thicknesses of 0.5 mm, 1.0 mm, and 1.5 mm, as follows:

Filtek Supreme XTE nanocomposite + Filtek Supreme Flow (3M) FR base (0.5, 1.0, 1.5 mm)
Filtek Supreme XTE nanocomposite + Fuji II LC (GC) RMGI base (0.5, 1.0, 1.5 mm)
Grandio Universal Nanohybrid + Grandio Flow (VOCO) FR base (0.5, 1.0, 1.5 mm)
Grandio Universal Nanohybrid + Fuji II LC (GC) RMGI base (0.5, 1.0, 1.5 mm)
Admira Fusion Nanohybrid Ormocer + Admira Fusion Flow (VOCO) FR base (0.5, 1.0, 1.5 mm)
Admira Fusion Nanohybrid Ormocer + Fuji II LC (GC) RMGI base (0.5, 1.0, 1.5 mm)

### 2.5 Control groups

The control groups included three restored teeth using only the corresponding packable nanocomposite (no base layer), and one healthy, non-restored tooth model:

Filtek Supreme XTE (without base)
Grandio Universal Nanohybrid (without base)
Admira Fusion Nanohybrid Ormocer (without base)
Healthy, intact tooth

**Figure 3:**
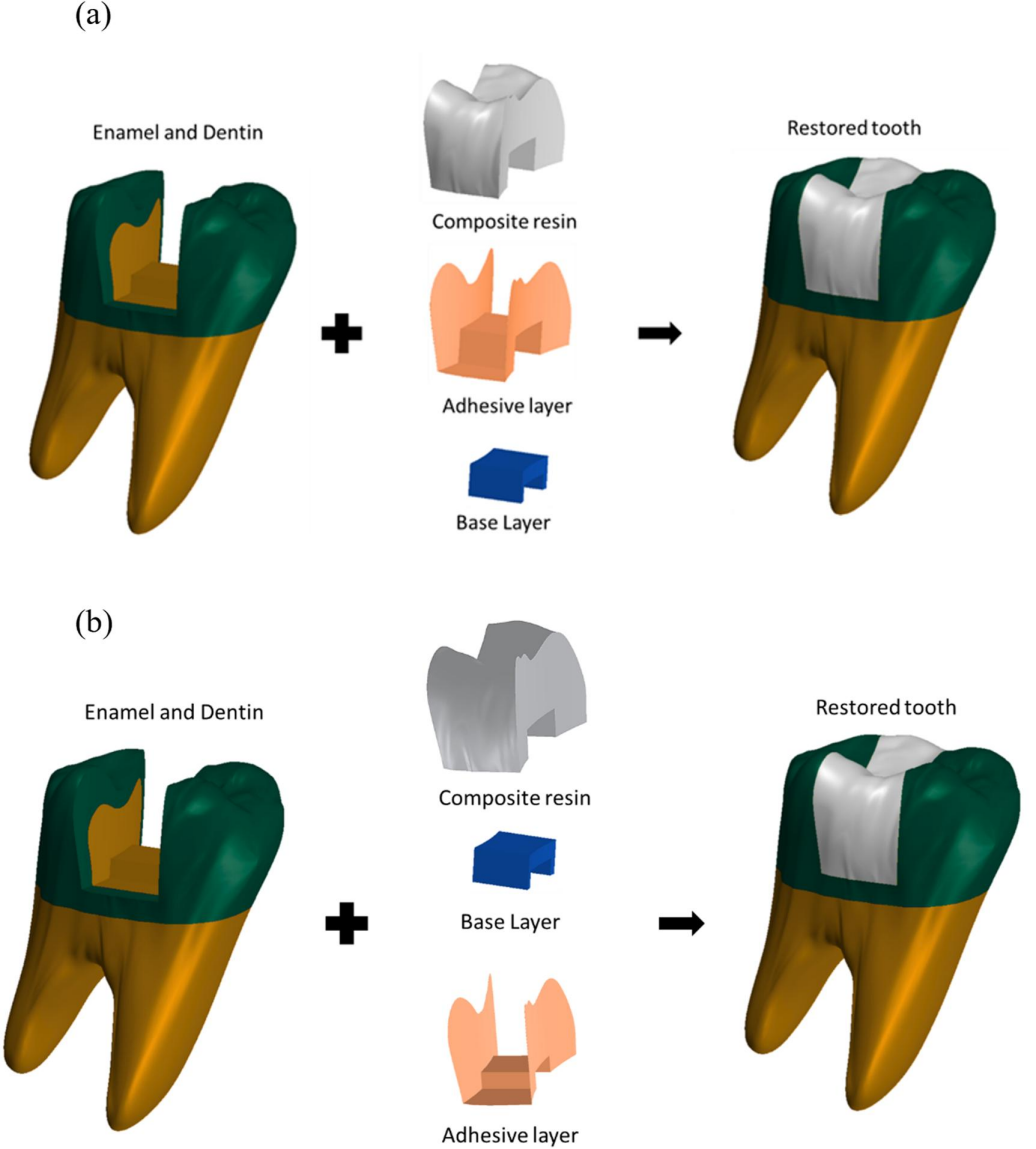
Design of the restoration method for the study groups. **(a)** Resin-modified glass ionomer- based. **(b)** Flowable resin-based.

In all groups utilizing flowable resin composite bases, a 0.005 mm adhesive layer was modeled beneath the base material. For groups with resin-modified glass ionomer bases, the adhesive layer was positioned above the base. The adhesive layer was explicitly modeled as a continuous solid layer and it was perfectly bonded to both tooth and restorative materials. No separate contact elements or frictional interfaces were defined at this locus; instead, a tie-constraint was used to ensure full continuity across the adhesive interfaces. This adhesive interface was modeled along all internal cavity walls across all test groups (Figure 3).

### 2.6 Experimental assumptions, boundary conditions, and parameter settings

In this study, all dental tissues and restorative materials were modeled as linear elastic, homogeneous, and isotropic. These assumptions are commonly adopted in dental finite element analyzes to reduce computational complexity while still capturing the main trends in stress distribution, particularly under static loading conditions. These assumptions are consistent with prior finite element studies in restorative dentistry (16, 27, 29–32). All interfaces between enamel, dentin, adhesive, base materials, and composite resins were modeled as perfectly bonded, without allowing debonding or sliding. This was implemented using tie constraints between the corresponding surfaces, ensuring full continuity of displacements and stresses across each interface. The corresponding mechanical properties and parameters applied to each material are summarized in Table 3.

**Table 3:** Physical and mechanical properties of the composite materials used.

Component	Modulus of elasticity (GPa)	Poisson's ratio
Enamel	84.1	0.3 (19)
Dentin	18.6	0.31 (19)
Pulp	0.23 × 10^−7^	0.3 (19)
Periodontal ligament	68.0 × 10^−3^	0.45 (19)
Food bolus	3.4	0.1 (27)
Clearfil SE adhesive layer	0.39	0.32 (25)
Fuji II GC glass ionomer	10.8	0.3 (26)
Grandio paste resin layer	17.9	0.31 (16)
Grandio fluid resin layer	6.85	0.31 (16)
Filtek supreme XTE paste resin layer	7.8	0.45 (16)
Filtek supreme XTE fluid resin layer	5.76	0.393 (16)
Admira Fusion paste resin layer	9.8	0.31 (16)
Admira Fusion fluid resin layer	3.29	0.34 (16)

### 2.7 Loading methods

**Figure 4:**
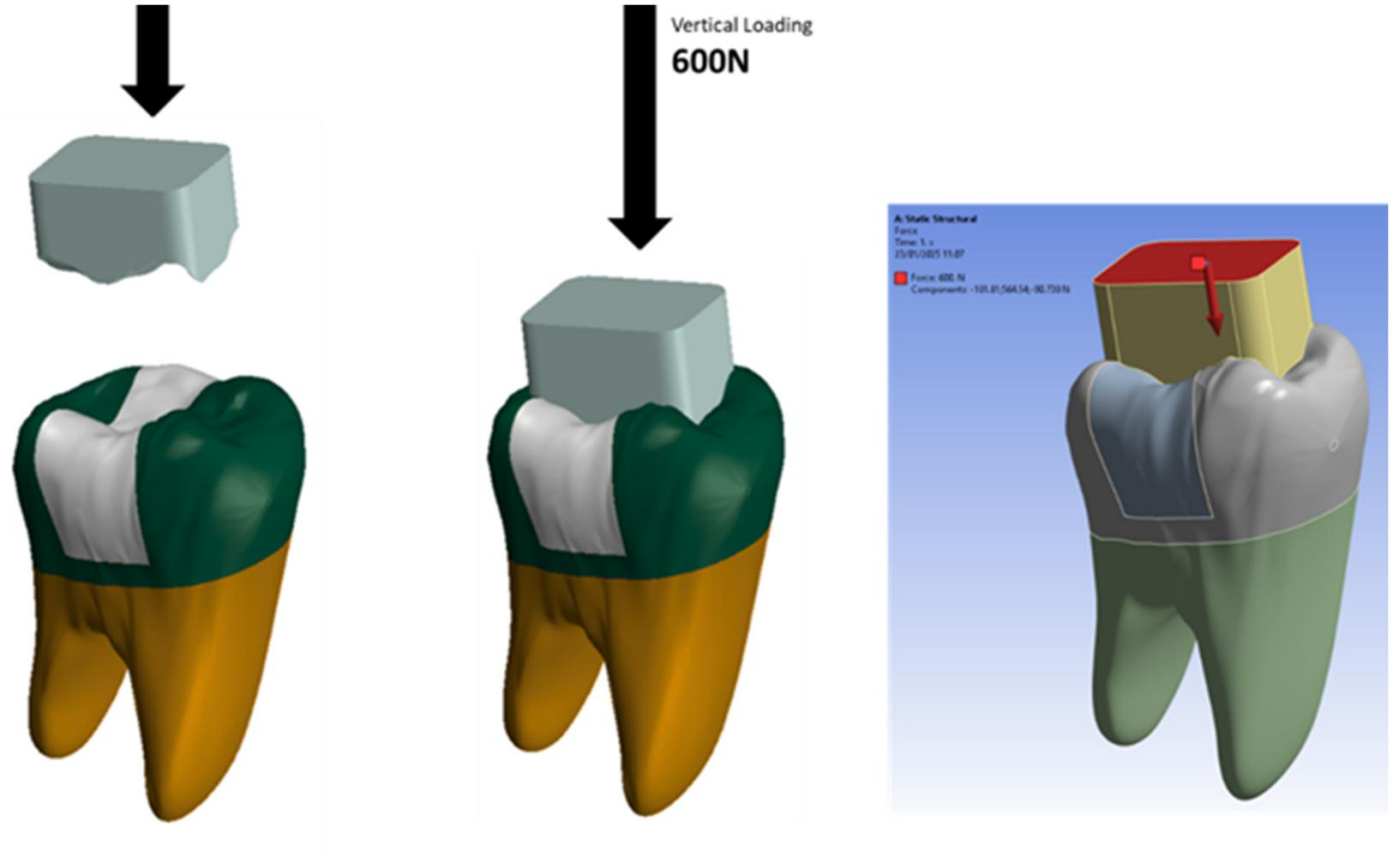
Load method design.

To replicate masticatory conditions, a food bolus model was digitally constructed, with its contact area extending from the central third of the occlusal surface to the marginal ridges of the tooth. Only a static vertical load of 600 N—representing the maximum bite force—was applied along the long axis of the tooth (26, 27). The load was distributed across the occlusal surface via the food bolus, simulating the final phase of the chewing cycle (Figure 4).

## 3 Results

Finite element analysis (FEA) revealed distinctive stress distribution patterns across the experimental and control models. Detailed maximum stress values for all restorative configurations are summarized in Table 4.

**Table 4:** Maximum stress values in restored teeth in MPa.

		Maximum stress (MPa)			
		Base layers thickness			
Composite resin	Base layer	0.5 mm	1 mm	1.5 mm	
Filtek supreme XTE paste resin	None	–	–	–	31.243
	Filtek Supreme XT flowable resin	31.072	23.560	31.247	–
	Fuji II GC glass ionomer	32.212	32.077	31.925	–
Grandio paste resin	None	–	–	–	30.313
	Grandio Flowable resin	30.845	23.560	31.281	–
	Fuji II GC glass ionomer	30.729	20.846	30.961	–
Admira fusion paste resin	None	–	–	–	31.117
	Admira Fusion flowable resin	31.536	25.890	32.031	–
	Fuji II GC glass ionomer	31.059	31.033	31.012	–
Healthy tooth	None	–	–	–	20.777

An intact, healthy tooth exhibited a uniform stress distribution throughout the enamel with a maximum von Mises stress of 20.777 MPa, establishing the biomechanical baseline for comparison. In models restored without a base layer, all composite systems generated significantly higher stress concentrations compared to the intact tooth. Filtek Supreme XTE exhibited the highest peak stress at 31.243 MPa, with concentrations localized primarily at the tooth-restoration interface, the proximal walls, and the pulpal floor.

The incorporation of an intermediate layer modified the stress distribution profiles. In models utilizing a flowable resin (FR) base, stress levels remained within similar anatomical regions but were moderately reduced; a slight decrease in stress was observed as base thickness increased. Conversely, restorations incorporating a resin-modified glass ionomer (RMGI) base demonstrated a substantial reduction in stress levels, an effect that became more pronounced with greater base thickness.

Among the unlined restorations, the Grandio composite yielded the lowest stress concentration at 30.313 MPa. When combined with an FR base, stress levels remained low and were localized similarly to the unlined restorations. However, at greater FR base thicknesses, stress concentrations began to emerge both above and below the base layer. The use of an RMGI base resulted in a marked reduction in stress regardless of the thickness applied.

## 4 Discussion

Extensive cavity designs, such as mesio-occluso-distal (MOD) preparations, significantly compromise the structural integrity of the tooth due to the substantial removal of enamel and dentin. Evidence indicates that MOD-prepared teeth may lose up to 60% of their original strength compared to intact teeth (21). Selecting an appropriate restorative approach is therefore critical, particularly given the challenges posed by limited gingival adhesion, insufficient polymerization depth, and shrinkage stresses during curing, which may lead to microleakage, recurrent caries, postoperative sensitivity, and imperfect restoration adaptation (10, 14). Finite element analysis (FEA) has become a widely accepted method for evaluating the mechanical behavior of restorative materials in complex cavity designs. Recent modeling efforts have focused on how various material combinations respond to occlusal forces and polymerization shrinkage in MOD restorations, offering critical insights into stress distribution and biomechanical performance (27–30).

To address these complications, one strategy involves the use of flowable base materials applied to the pulpal floor, leveraging their adaptability and elastic properties to absorb stresses and improve marginal adaptation (16). In this context, the present study aimed to investigate how different packable nanocomposites, combined with two base materials applied at varying thicknesses, affect the stress distribution in direct MOD restorations.

Overall, our findings revealed that stress distribution in the sound tooth was homogeneous throughout the enamel, with no critical areas of stress concentration. This is consistent with previous reports indicating that an unrestored tooth dissipates occlusal forces uniformly across the hard dental tissues (26). In contrast, extensive Class II restorations altered the biomechanical response of the tooth–restoration complex. Under identical loading conditions, clear differences in von Mises stress distribution were observed among the restorative designs analyzed. Therefore, the null hypothesis—that no differences in stress distribution would be found among the various designs—was rejected.

Restorations incorporating a base material consistently exhibited lower von Mises stress levels compared with restorations composed solely of composite resin. The use of a liner resulted in a reduction in overall rigidity, supporting a more favorable biomechanical behavior within the restored tooth. These findings are consistent with previous studies, which similarly reported improved stress distribution and enhanced mechanical performance when intermediate base materials were employed (5, 8, 29, 30).

The following sections provide a detailed comparative analysis of each variable examined in this study.

### 4.1 Base thickness

Like other glass ionomer cements, resin-reinforced, light-curing Fuji II LC glass ionomer cement is based on the reaction between silicate glass and polyacrylic acid to form a hardening matrix. Light polymerization accelerates this reaction, reducing the setting time (33). Compared to packable resins, fluid resins have a lower filler load and a higher proportion of diluent monomers, making them less viscous (21). Because of these properties, they are currently the most widely used coating materials by dentists.

This study found that restorations coated with RMGI exhibited significantly lower von Mises stress than those coated with FR or left uncoated. This is likely due to the fact that RMGI has a lower elastic modulus. Additionally, RMGI undergoes expansion due to water absorption after setting. According to Hooke's law, contraction stress [σ] is directly related to volumetric contraction (*Δ*V) and elastic modulus (E). Therefore, materials with lower E generate lower σ for the same shrinkage (34). Furthermore, recent studies have shown that RMGIs have zero volumetric shrinkage due to their acid-base reaction and that their unique inner layer cushions the stresses of the underlying composite (1). This explains why restorations with RMGI showed much lower stress. Being more flexible, they act as shock absorbers and distribute the load more smoothly, reducing stress peaks at the dentin-restoration interface. These results coincide with those of Di Lauro et al., who demonstrated that hybrid glass ionomer layers under bulk polymer composites produced stress distributions similar to intact teeth (6). However, a notable discrepancy exists with the study by Ausiello et al., who concluded that conventional glass ionomer cement was ineffective in reducing stress within Class II MOD cavities (27). This divergence can be attributed to critical differences in mesh architecture and finite element formulation. In the aforementioned study, linear 4-node tetrahedral elements (CTETRA) were utilized, which rely on first-order interpolation functions. In computational mechanics, these elements are inherently stiff and susceptible to shear locking; furthermore, they assume a constant strain within the element volume (28). Consequently, they tend to underestimate stress magnitudes in regions with high gradients, such as the pulpal floor or adhesive interfaces. In contrast, our research implemented quadratic 10-node tetrahedral elements, which incorporate mid-side nodes along each edge. This configuration enables second-order interpolation functions where strain varies linearly across the element, capturing localized stress concentrations with higher fidelity without necessitating a prohibitively dense mesh. Moreover, the ability of quadratic edges to represent curved surfaces allows the model to conform with superior geometric precision to the complex anatomical morphology derived from 3D scanning, surpassing the “faceted” representation characteristic of 4-node elements (28). Additionally, the elastic modulus assigned to the base material differs significantly (8.0 GPa in the cited study vs. 10.8 GPa in our model). Factors such as this disparity in relative stiffness and the uniformity of the base layer thickness are decisive influences on the damping capacity and stress redistribution patterns of the restorative system. Furthermore, the biomechanical behavior observed in our model is consistent with more recent reports by Ausiello et al., regarding Class I (29) and Class II MO cavities (30), where the application of glass ionomer as a base layer was validated to reduce internal stresses. Although the numerical modeling strategy in those studies maintained similarities to their previous work, the apparent contradiction is primarily explained by the complex interaction between the cavity configuration factor (C-factor) and the total volume of restorative material. In deep MOD cavities, optimizing the base layer thickness and employing higher-order elements are critical requirements for accurately detecting stress attenuation and ensuring structural stability under functional loading. In contrast, Mahmoudi-Yamchi et al. evaluated deep margin elevation in endocrowns and reported that FRs and RMGIs significantly reduced von Mises stress in tooth structures without altering stress distributions between base materials (19). This differs from our observation regarding the specific benefit of RMGI. This is probably due to the different boundary conditions, geometries and restorative materials used.

In a related study, Matuda et al. (31) used finite element analysis to evaluate premolar Class II cavities restored with resin composites, observing that stress concentration patterns were strongly influenced by cavity shape and composite configuration. Although their model did not include base layers, their results partially align with our unlined and FR-based groups in terms of stress distribution trends. The main differences likely arise from variations in the boundary conditions, load direction, and mesh complexity. Taken together, our model is broadly consistent with the existing FE literature on adhesive restorations (27–30), but provides additional insight by directly comparing base materials and thicknesses under standardized conditions—factors that have been less explored in prior analyses. This comparative perspective enhances the interpretability and relevance of our results within the wider field of restorative biomechanics.

### 4.2 Coating thickness

This study evaluated base thicknesses of 0.5, 1.0, and 1.5 mm for both resin-modified glass ionomer (RMGI) and flowable resin (FR). Finite element analysis demonstrated a marked reduction in von Mises stress with increasing RMGI base thickness, whereas only a modest decrease was observed as FR base thickness increased. In contrast, restorations incorporating Ormocer-based bases exhibited an opposite trend, with stress concentrations increasing as base thickness increased. This behavior may be attributed to the higher elastic modulus of Ormocer materials compared with the other base materials evaluated.

The moderate stress reduction observed with FR bases may be attributed to their relatively higher elastic modulus combined with polymerization shrinkage, which typically ranges from 2% to 5% (20). As the thickness of these materials increases, the cumulative shrinkage-induced stress may remain substantial, thereby limiting their stress-buffering effectiveness. Consequently, FR bases may exhibit a reduced damping capacity, favoring stress accumulation at internal line angles and material interfaces due to stiffness mismatches and shrinkage-related strains (33).

**Figure 5:**
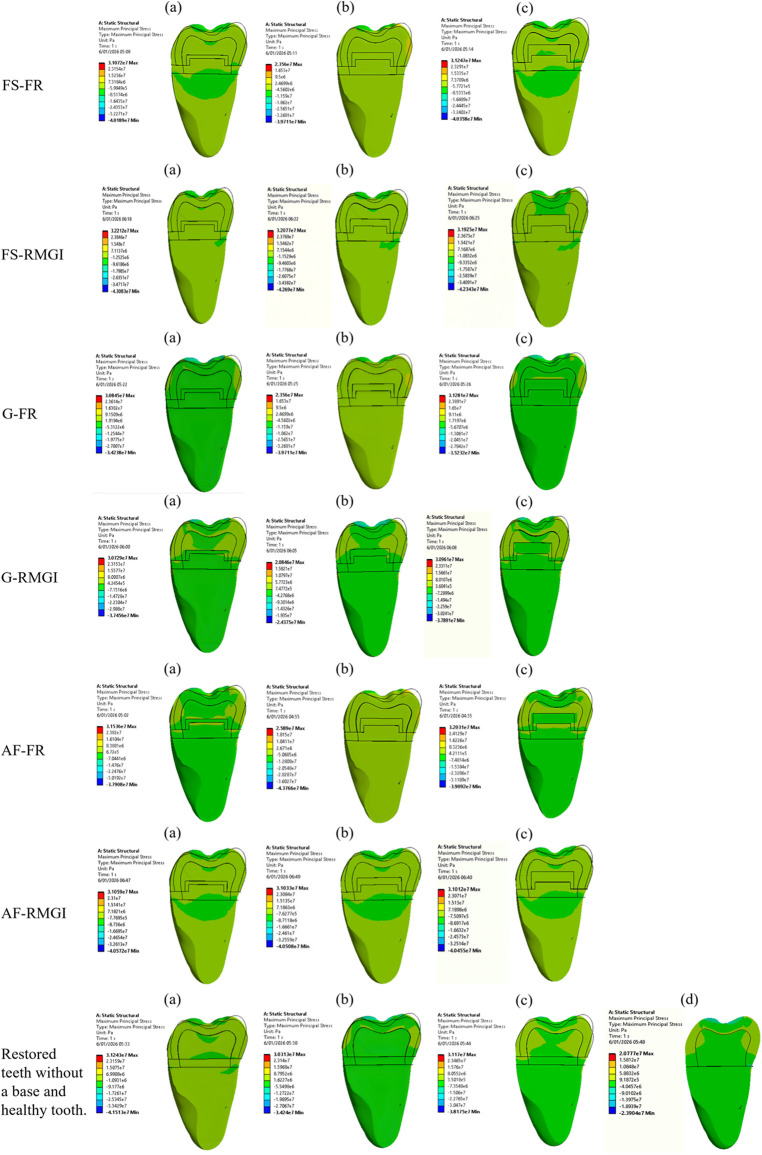
Von misses stress distribution after loading effect of 600 N in a buccolingual cross-sectional view. Teeth restored with Filtek Supreme (FS) with FR base and RMGI at thicknesses **(a)** 0.5 mm, **(b)** 1 mm, **(c)** 1.5 mm. Teeth restored with Grandio (G) with FR base and RMGI at thicknesses **(a)** 0.5 mm, **(b)** 1 mm, **(c)** 1.5 mm. Teeth restored with Admira Fusion (AF) with FR base and RMGI at thicknesses **(a)** 0.5 mm, **(b)** 1 mm, **(c)** 1.5 mm. The control groups **(a)** tooth restored with Filtek Supreme only, **(b)** tooth restored with Grandio only, **(c)** tooth restored with Admira Fusion only and **(d)** healthy tooth.

These findings are consistent with those of Kaisarly et al., who reported that thinner FR bases applied using incremental techniques resulted in more favorable stress distribution compared with bulk-fill approaches (17). Conversely, Jung et al. demonstrated that both RMGI and FR bases significantly reduced polymerization-induced marginal stresses, irrespective of base thickness, when compared with restorations placed without a base (26) (Figure 5).

From a clinical standpoint, the present findings suggest that resin-modified glass ionomer (RMGI) liners of 1.0–1.5 mm thickness provide the most favorable stress distribution in Class II MOD restorations, particularly when used beneath Grandio and Admira Fusion composites. Thicker RMGI layers were associated with a more pronounced reduction in stress at the dentin–restoration interface, whereas increasing the thickness of low-viscosity resin bases offered only modest additional benefit and, in some cases, tended to increase localized stresses. Within the limitations of a purely computational study, clinicians may therefore consider using an RMGI liner of approximately 1.0–1.5 mm in deep proximal boxes when restoring large Class II cavities with nanofilled composites (3, 21). However, these recommendations should be interpreted with caution until confirmed by long-term clinical and laboratory studies.

### 4.3 Restorative material

The three cutting-edge packable materials in this study were a Filtek Supreme XT nanocomposite; a nanohybrid, Grandio; and an Ormocer, Admira Fusion. Restorations coated with Admira Fusion exhibited higher stress than those restored with the other materials. The data obtained in this study confirm the correlations that exist between mechanical properties and maximum stresses. A material with increased stiffness causes a notable increase in residual stresses (5). In this study, the stresses transmitted by Admira Fusion at the tooth-restoration interface and on the veneering material are mainly due to its relatively high stiffness. Therefore, the greater the stiffness of the material, the lower its ability to mitigate residual stress through deformation. As for its other property, Poisson's ratio, if it were too high, lateral deformation would occur on the buccal-lingual walls of the tooth, increasing stresses in this area. However, the Poisson's ratios in the materials studied were similar, so they had very little effect in terms of this magnitude. Bansal et al. reported something similar: a less rigid material (Activa Bioactive) exhibited greater deformation and absorbed more stress internally compared to a more rigid material (EverX) (24). On the other hand, uncoated restorations made with Filtek Supreme XT generated the highest stress concentrations across the entire tooth-restoration interface, which is consistent with the observations of Tavangar et al., where the highest stress values were obtained in uncoated hybrid composites (7). In contrast, Grandio and Admira Fusion restorations without lining showed moderate to low stress, suggesting a lower modulus of elasticity and polymerization shrinkage.

Several limitations of this study should be considered. Dental tissues and restorative materials were modeled as linear elastic, homogeneous, and isotropic, which, although common in dental finite element analysis, does not fully represent their anisotropic and time-dependent behavior. In addition, polymerization shrinkage and the associated contraction stresses of resin-based materials were not simulated, despite their known influence on stress development at the tooth–restoration interface. The loading protocol was limited to a single static vertical load applied along the tooth's long axis, without accounting for oblique forces, cyclic loading, or thermomechanical aging that characterize intraoral conditions. Furthermore, the numerical model was not directly validated against experimental measurements, nor was a formal sensitivity analysis performed. Accordingly, the findings should be interpreted as idealized and comparative rather than as absolute clinical stress values. However, the observed stress distribution patterns and the relative influence of the base materials are consistent with trends reported in previous finite element and *in vitro* studies. Future research incorporating more advanced material models, complex loading scenarios, and combined experimental–numerical validation would further enhance the clinical relevance of these findings. Such advancements would not only refine FEA methodologies but also support the development of evidence-based protocols for restorative decision-making in structurally compromised posterior teeth.

## 5 Conclusion

The use of an intermediate base material in Class II MOD restorations proves to be an effective strategy for reducing polymerization shrinkage-induced stresses. Among the evaluated base materials, thicker resin-modified glass ionomer (RMGI) liners demonstrated superior stress distribution capabilities compared to flowable resin composite bases. Furthermore, when used in conjunction with a liner, the Grandio and Admira Fusion restorative systems demonstrated superior biomechanical behavior compared to Filtek Supreme XTE. These findings suggest that such combinations may enhance the long-term durability and clinical success of posterior composite restorations by optimizing stress distribution within the tooth-restoration complex.

## Data Availability

The original contributions presented in the study are included in the article/Supplementary Material, further inquiries can be directed to the corresponding author.
